# Many paths to one goal: Identifying integrated rice root phenotypes for diverse drought environments

**DOI:** 10.3389/fpls.2022.959629

**Published:** 2022-08-22

**Authors:** Jenna E. Fonta, Phanchita Vejchasarn, Amelia Henry, Jonathan P. Lynch, Kathleen M. Brown

**Affiliations:** ^1^Intercollege Graduate Degree Program in Plant Biology, Huck Institutes of the Life Sciences, Penn State University, University Park, PA, United States; ^2^Department of Plant Science, The Pennsylvania State University, University Park, PA, United States; ^3^Rice Department, Ministry of Agriculture, Ubon Ratchathani Rice Research Center, Ubon Ratchathani, Thailand; ^4^Rice Breeding Innovations Platform, International Rice Research Institute (IRRI), Los Baños, Philippines

**Keywords:** rice, roots, metaxylem, drought stress, root plasticity, integrated phenotype, *Oryza sativa* (L.)

## Abstract

Drought is a major source of yield loss in the production of rice (*Oryza sativa* L.), and cultivars that maintain yield under drought across environments and drought stress scenarios are urgently needed. Root phenotypes directly affect water interception and uptake, so plants with root systems optimized for water uptake under drought would likely exhibit reduced yield loss. Deeper nodal roots that have a low metabolic cost per length (i.e., cheaper roots) *via* smaller root diameter and/or more aerenchyma and that transport water efficiently through smaller diameter metaxylem vessels may be beneficial during drought. Subsets of the Rice Diversity Panel 1 and Azucena × IR64 recombinant inbred lines were grown in two greenhouse and two rainout shelter experiments under drought stress to assess their shoot, root anatomical, and root architectural phenotypes. Root traits and root trait plasticity in response to drought varied with genotype and environment. The best-performing groups in the rainout shelter experiments had less plasticity of living tissue area in nodal roots than the worst performing groups. Root traits under drought were partitioned into similar groups or clusters *via* the partitioning-around-medoids algorithm, and this revealed two favorable integrated root phenotypes common within and across environments. One favorable integrated phenotype exhibited many, deep nodal roots with larger root cross-sectional area and more aerenchyma, while the other favorable phenotype exhibited many, deep nodal roots with small root cross-sectional area and small metaxylem vessels. Deeper roots with high theoretical axial hydraulic conductance combined with reduced root metabolic cost contributed to greater shoot biomass under drought. These results reflect how some root anatomical and architectural phenes work in concert as integrated phenotypes to influence the performance of plant under drought stress. Multiple integrated root phenotypes are therefore recommended to be selected in breeding programs for improving rice yield across diverse environments and drought scenarios.

## Introduction

Drought is a major constraint to rice production, particularly in rain-fed systems. Over 3.5 billion people consume at least 20% of their daily calories from rice, and many of them are poor and rely on rice as both an income and food source. Improving rice drought tolerance is therefore of utmost importance. Root phenotypes are a good target for improving drought tolerance in crops since they affect water interception, uptake, and transport to shoot tissues (Lynch, 2021).

The rice root system is composed of a small primary root system with nodal roots that emerge at successive nodes. The internal structure of rice roots consists of outer epidermal cell layers, a cortex that contains an outer suberized cell layer but is comprised primarily of aerenchyma (hollow air spaces), a suberized endodermis, and a central cylinder containing xylem and phloem tissues. Root anatomical traits vary significantly among rice genotypes (Terashima et al., 1987; Champoux et al., 1995; Kondo et al., 2000; Uga et al., 2008, 2009; Vejchasarn et al., 2016), making it possible to relate their variation to the performance of plant. Many root traits are also responsive to environmental signals, including drought, and nutrient availability (Gowda et al., 2011; Vejchasarn et al., 2016; Hazman and Brown, 2018), and the plasticity of root phenotypes is under genetic control (Nicotra and Davidson, 2010) in many crops, including maize (Schneider et al., 2020a,b) and rice (Niones et al., 2013, 2015). Both stable traits and their plasticity are therefore potential targets for breeding to improve drought tolerance in rice.

During a soil drying event, soil water gradually dries down from the topsoil, often leaving more water available at depth. Deeper rooting is therefore beneficial under most drought scenarios, including in rice production systems (Gowda et al., 2011; Uga et al., 2013; Arai-Sanoh et al., 2014). In maize, traits such as steeper root growth angle, fewer nodal roots, greater lateral root branching at depth, and metabolically cheaper roots have been proposed to increase deep water capture (Lynch, 2015, 2021). Maize genotypes with fewer nodal roots had deeper rooting and more biomass and yield under drought (Gao and Lynch, 2016). Anatomical traits that reduced metabolic cost per unit root length improved performance under drought and low nutrient stress in maize (Chimungu et al., 2014a,b; Saengwilai et al., 2014a; Lynch, 2015; Castañeda et al., 2018) and common bean (Strock et al., 2018, 2019; Strock and Lynch, 2020). Steeper root growth angle has been employed for mitigating drought stress in rice (Uga et al., 2013). More root aerenchyma formation led to more lateral root production and greater biomass (Niones et al., 2013) and yield (Niones et al., 2012) in rice under drought. Roots with large diameters have been shown to improve drought tolerance in rice by increasing soil penetration ability (Materechera et al., 1992; Nguyen et al., 1997; Clark et al., 2008), which is limited by the hardpan in flooded fields and by dry compacted soils in upland fields. Large diameter roots can remain metabolically inexpensive if they have a greater proportion of aerenchyma, which does not diminish penetration ability in rice roots (Striker et al., 2007).

Variation in root xylem phenotypes can also affect drought tolerance due to their effect on water transport along root axes. According to the Hagen–Poiseuille equation, axial hydraulic conductance through vessels assumed to be cylindrical pipes can be estimated based on the size and number of each vessel (Tyree and Ewers, 1991). Roots with reduced axial hydraulic conductance *via* smaller or fewer vessels would transport less water axially, conserving soil water resources and keeping the growing root tip hydrated. Smaller xylem vessels and reduced hydraulic conductance are beneficial under drought in wheat seedlings (Richards and Passioura, 1989) and in maize (Klein et al., 2020). Xylem diameter is reduced in response to drought in rice (Henry et al., 2012) but has yet to be related to performance under drought. In agroecosystems where deep moisture is available, optimal xylem phenotypes are likely those that increase water use efficiency with minimal effects on shoot growth and yield (Lynch, 2021).

The ability of a phenotype to change in response to environmental stimuli is termed plasticity. Root phenotypic plasticity has been shown to be beneficial in numerous drought scenarios in rice (Suralta et al., 2010, 2016; Henry et al., 2012; Sandhu et al., 2016), and plasticity of root anatomical and architectural phenotypes has been observed in rice. However, greater root architectural plasticity is not always associated with greater yield stability across diverse drought stress treatments (Sandhu et al., 2016). The temporal dynamics of both the imposed stress and the trait response determine the benefits of plasticity, since some aspects of the root phenotype respond more slowly to environmental change than others (Schneider and Lynch, 2020). The benefits and trade-offs of root phenotypic plasticity must, therefore, be carefully evaluated across environments and drought stress scenarios. It is likely that optimal degrees of plasticity will vary depending on the trait.

Root phenotypes do not exist in isolation, and their individual effects on whole plant productivity are influenced by other root and shoot phenotypes. This interplay among phenes (elements of the phenotype, York et al., 2013) determines the performance of each integrated phenotype. Integrated phenotypes are more informative for understanding the performance under drought and/or low fertility than individual root phenes considered separately due to phene interactions (Miguel et al., 2015; York and Lynch, 2015; Sandhu et al., 2016; Strock et al., 2019, 2020; Klein et al., 2020). Multiple integrated phenotypes may be effective in the same environment, as shown *in planta* in maize under drought stress (Klein et al., 2020) and *in silico* in common bean under suboptimal nitrogen and phosphorus availability (Rangarajan et al., 2018). We propose that multiple integrated root phenotypes will be effective in maximizing the performance of rice in drought environments. We hypothesize that root systems that can reduce root metabolic cost *via* smaller diameter roots and greater aerenchyma formation and modulate water uptake with smaller metaxylem vessels should grow deeper roots and transport water more efficiently, resulting in better performance under drought stress.

To identify root phenotypes associated with improved performance under drought stress, we grew diverse rice genotypes in four experiments, two in greenhouses, and two in rainout shelters in rice-growing areas and imposed drought stress during vegetative or panicle initiation stages. We compared drought responses of root anatomical and architectural phenotypes and identified integrated root phenotypes common among environments. From these results, we show that multiple integrated phenotypes are effective in conferring drought tolerance in rice within and across environments.

## Materials and methods

### Plant materials and growth conditions

Root phenotypes were assessed in drought experiments in greenhouses at Penn State University, University Park, PA (40° 48 7.4 *N*, 77° 51 46.5 W), in rainout shelters at the International Rice Research Institute (IRRI), Los Baños, Philippines (14° 10 12.2 *N*, 121° 15 38.2 E), and in rainout shelters at the Ubon Ratchathani Rice Research Center (URRC), Ubon Ratchathani, Thailand (15° 19 56.9 *N*, 104° 41 9.6 E). Genotypes from three different populations were used in this study to determine whether root responses were consistent among different genetic backgrounds. Fourteen genotypes from the Rice Diversity Panel 1 (RDP1) from the *indica* and *tropical japonica* subpopulations, chosen for contrasts in aerenchyma formation, were grown at Penn State (RDP1 PSU) from April to May 2016 and at IRRI (RDP1 IRRI) from January to May 2016 (dry season). Thirty-two randomly selected genotypes from an Azucena (*tropical japonica*) × IR64 (*indica*) recombinant inbred line (RIL) population (Guiderdoni et al., 2006) were grown at Penn State (RILs PSU) from April to December 2017. All 140 genotypes in the *tropical japonica* and *aus* subpopulations within RDP1 were grown at URRC (RDP1 URRC) from September to December 2018 (Supplementary Table 1).

In both the RDP1 PSU and the RILs PSU experiments, plants were arranged in a complete block design in plastic mesocosms (15 cm diameter × 1.2 m tall) lined with polyethylene liners. A growth medium of 40% v/v medium sand (0.3–0.5 mm), 40% v/v vermiculite (Griffin Greenhouse Supplies, Morgantown, PA, United States), 5% v/v perlite (Griffin Greenhouse Supplies, Morgantown, PA, United States), 15% v/v sifted field soil (Hagerstown silt loam; 64% silt, 21% clay, 15% sand; fine, mixed, semi-active, medic Typic Hapludult), and solid slow-release fertilizer pellets at 3.2 g/L (Osmocote Plus, 15% N, 9% P_2_O_5_, 12% K_2_O, 1% Mg, 6% S, 0.02% B, 0.05% Cu, 0.4% Fe, 0.06% Mn, 0.02% Mo, 0.05% Zn, 3–4 month release) was used for ease of washing from root tissue while maintaining some structure and ion exchange capacity. Seeds were de-hulled, sterilized with 10% (v/v) NaOCl, rinsed with deionized water, and planted directly into mesocosms at field capacity (soil media flooded and let drain for 2–4 h). In the RILs PSU experiment, wire single-mesh baskets (14.3 cm diameter, 13 cm tall, Winco, NJ, United States) were placed into the top 10 cm of the mesocosm for the measurement of nodal root angle, and seeds were planted directly into the media-filled baskets. Temperature was maintained at 25–28°C, and supplemental LED lights [200 μmol m^–2^ s^–1^ photosynthetically active radiation (PAR)] were used for a 14-h day length when ambient light was less than 600 μmol m^–2^ s^–1^ PAR. Plants were watered twice daily with deionized water. Two weeks after germination, the drought treatment was imposed by withholding water. Volumetric water content was measured biweekly using time-domain reflectometry (TDR100, Campbell Scientific, Logan, UT, United States) with probes inserted 30.5 cm from the top and 30.5 cm from the bottom of the mesocosms to measure surface soil drying, as well as to monitor the moisture in deeper soil.

In the RDP1 IRRI experiment, plants were arranged in a randomized complete block design with four plot replications per genotype. The drought treatment was applied in a field with a rainout shelter automated to cover the plots during rain events, and plots assigned to the control treatment were grown in an adjacent field. The soil type at this site was an Isohyperthermic Typic Hapludalf (Soil Survey Staff, 1999). At midday during the week of root sampling, air temperature ranged from 32°C to 34°C, and humidity ranged from 47 to 55%. Seeds were planted in a seed bed, and 3 weeks after germination were hand-transplanted to plots with three 3 m long rows per plot and 0.2 m between hills. Two weeks after transplanting, the drought treatment field was drained to impose stress. Basal fertilizer (50-50-50) application occurred at 36 DAS, and imidacloprid (Provado, Bayer Crop Science, St. Louis, MO, United States) was applied at 43 DAS to control thrips. Tensiometers (Soilmoisture Equipment Corp, Santa Barbara, CA, United States) were used to measure soil moisture at 30 cm below the soil surface throughout the drought period. Soil water potential ranged from -20 to -50 kPa in the drought treatment plots, which were re-watered at 74 and 84 DAS when soil water potential reached -50 kPa, and the well-watered treatment remained flooded throughout the experiment.

For the RDP1 URRC experiment, plants were arranged in a randomized complete block design with two treatments and three randomized blocks per treatment with one plant per genotype per block, for a total of three biological replicates per genotype per treatment. The drought and well-watered treatments were each grown in cement troughs (14.78 m length × 1.80 m width × 0.53 m height, soil depth 0.38 m) with a movable rainout shelter for cover, while the well-watered treatment was continuously flooded. Genotypes were direct-seeded with 20 × 20 cm spacing between plants. The soil texture was sandy (74% sand, 15% silt, and 11% clay), and plots remained flooded in both treatments until 5 weeks after emergence (panicle initiation) when water was drained from the drought treatment. Water-soluble fertilizers (15-0-0; 0-0-50; 20-20-20 + 200 ppm B, 1,000 ppm Fe, 500 ppm Mn, 150 ppm Zn, 110 ppm Cu, 70 ppm Mn; 4% Fe, 4% Mn, 1.5% Cu, 0.5% B, 1.5% Zn, 0.1% Mo) were applied 3 times per week until 9 days before drought was imposed. General insecticide beta-cyfluthrin (Folitec, Bayer Crop Science, St. Louis, MO, United States was used twice during the experiment, and *Paecilomyces lilacinus* was applied before planting to control for root-knot nematode. Air temperatures ranged from 24 to 30°C and humidity from 63 to 95%.

### Plant sampling and phenotyping

In the RDP1 PSU and the RILs PSU experiments, plants were harvested 4 weeks after the onset of drought stress, 6 weeks after emergence. At harvest, tiller number was counted, and shoots were excised for shoot dry biomass determination. The root systems and media mix contained within the plastic liner were laid horizontal and pulled from the pots, and the liner was cut open to expose the roots and growth medium. The maximum depth of the root system was recorded, and the remaining growth medium was gently washed from the root system. Clean root systems were preserved in bags of 70% v/v ethanol in water for later processing. Nodal root number was counted, and tissue from six roots with a length greater than half the length of the total root system was collected at 10 and 20 cm from the root tip. Root segments were placed in histocaps and dried in a critical point drier (Leica EM CPD 300, Leica Microsystems Inc., Buffalo Grove, IL, United States). High-resolution images of root transverse cross-sections were taken with laser ablation tomography (Hall et al., 2019). Mipar^®^ software was used to assess root cross-sectional area (RXSA), stele area (TSA), cortical area (TCA), median metaxylem vessel area (MXA), number of metaxylem vessels (MXV), and aerenchyma area (AA) from root images (Table 1). Percent aerenchyma area (percAA) was calculated as: AA/TCA*100. Living tissue area (LTA) was calculated as: RXSA – AA – MXA. Phenotypic plasticity was calculated as the relative difference in the genotype mean of the trait under drought treatment (D) compared to well-watered treatment (W): (x̄_*D*_ – x̄_*W*_)/ x̄_W_*100

**TABLE 1:** Description of trait abbreviations.

Trait	Description
RXSA	Root cross-sectional area (mm^2^)
LTA	Living tissue area (mm^2^)
TSA	Total stele area (mm^2^)
TCA	Total cortical area (mm^2^)
AA	Aerenchyma area (mm^2^)
percAA	Aerenchyma area (%)
MXA	Mean metaxylem vessel area (mm^2^)
MXV	Metaxylem vessel number
MXA.RXSA	Mean metaxylem vessel area/Root cross-sectional area
MXA.TSA	Mean metaxylem vessel area/Stele area
MXV.RXSA	Metaxylem vessel number/Root cross-sectional area (mm^–2^)
MXV.TSA	Metaxylem vessel number/Stele area (mm^–2^)
Biomass	Shoot dry biomass (g)

In the RDP1 IRRI experiment, the drought treatment plot was re-watered 1 day before root sampling. Three plants per plot were sampled at 53 days after transplanting. Tiller number was counted, and shoots were excised and dried. Root crowns were removed from the soil with a shovel to capture ∼20 cm root length below the soil surface and washed with water before being placed in 70% ethanol in water. Three between-hill soil cores were taken per plot (4 cm diameter × 40 cm deep). Soil cores were divided into 10 cm segments, and root tissue was washed from soil using a fine mesh strainer. Roots were then scanned on a flat-bed scanner (Epson V700 Photo, Epson America Inc., Los Alamitos, CA, United States), and WinRhizo Pro 2019 (Regent Instruments Inc., Quebec, Canada) was used to measure the total nodal root length per sample, where nodal roots were distinguished from other root classes as having a diameter of > 0.05 cm. The proportion of deep roots was calculated as the proportion of total root length in samples from 20 to 40 cm below the soil surface compared to the total root length in the soil core. Nodal root number was counted, and nodal root anatomy samples were taken from six representative roots at 5–10 cm from the root base. Samples were stored in 70% ethanol in water and shipped to Penn State University. Anatomical root phenotyping was carried out using the same methods as in the RDP1 PSU and the RILs PSU experiments.

In the RDP1 URRC experiment, the drought treatment plot was re-watered 2 days before root sampling (83 DAS). Tiller number, plant height, leaf rolling score, leaf water potential, and soil plant analysis development chlorophyll meter (SPAD) were measured on each plant. Shoot tissue was removed for dry biomass determination, and the root crown was excavated using a 20 cm diameter × 20 cm height steel monolith ring. Nodal root angle was calculated after washing the root crown by using root length as the hypotenuse and the cylinder height (7.5 cm) as the opposite side to determine the angle of root emergence from the crown. The number of deep roots was defined as those with a nodal root angle of > 30° from the soil surface. Root systems were preserved in 70% ethanol. Root anatomy samples were taken at 5–8 cm from the root base and again preserved in 70% ethanol. Transverse root cross-sections were taken using a vibratome (Leica VT1000 S, Leica Microsystems Inc., Buffalo Grove, IL, United States), and 12 cross-sections were imaged under a macro-zoom stereo microscope (MVX 10, Olympus, Japan). Anatomical phenotypes as mentioned above were measured with GIMP v 2.8 (GNU Image Manipulation Program; The GIMP Development Team, 2019) and Image J software (Schneider et al., 2012).

### Data analysis

All data analysis and visualizations were conducted in R v3.5.3 (R Core Team, 2016). Two- and three-way ANOVA along with effect size (η^2^, %) calculations (*DescTools* package; Signorell, 2020) were used to test the significance of factor effects on root phenotypic variation. Correlations between root phenotypes were calculated and visualized with the *corrplot* package (Murdoch and Chow, 1996; Friendly, 2002). Means and confidence intervals for each phenotype within treatment and experiment were calculated using the *Rmisc* package (Hope, 2013). Bulked segregant analysis was conducted by dividing genotypes within each experiment into groups based on the reduction in shoot biomass under drought. Genotypes with mean biomass greater than one standard deviation from the mean under well-watered conditions were filtered out, and the remaining genotypes were grouped into best and worst groups with less than or greater than 50% biomass reduction in drought. Boxplots for phenotypes within the groups were generated with the *ggplot2* package (Wickham, 2016). Multiple comparison tests on root phenotypes between experiments and performance groups were conducted using Tukey’s honestly significant difference (HSD) method with the *agricolae* package (de Mendiburu, 2020). Genetic coefficients of variation (GCV) were calculated within and among genotypes for each phenotype, experiment, and treatment as follows: GCV = σ/μ * 100, where σ is the standard deviation of the phenotype within individual genotypes or among all genotypes, respectively, and μ is the population mean of the phenotype.

Using root phenotypes under drought stress, individuals within each experiment were grouped by similar root phenotypes using partitioning around medoids (PAM) clustering with the R packages *factoextra* (Kassambara and Mundt, 2019) and *cluster* (Maechler et al., 2019). PAM clustering is a type of K-medoids clustering that groups objects to minimize dissimilarity. As in the bulked segregant analysis, individuals with biomass means within one standard deviation of the mean shoot biomass under well-watered conditions for each experiment were used for clustering. Within each experiment, data were centered and scaled before clustering. Optimal numbers of clusters were determined by testing both sum of squares and average silhouette scores, which are the average measures of similarity of an individual to its cluster compared to other clusters, meaning a larger value indicates better clustering. Silhouette plots were generated using *factoextra* (Kassambara and Mundt, 2019) to observe the similarity of individuals to others within the same cluster. The first two principal component loadings were plotted using *factoextra* (Kassambara and Mundt, 2019) to observe the separation of clusters. Mean phenotype values within each cluster were calculated and ranked highest to lowest compared to the other clusters in the experiment. Root phenotype value rankings per cluster (1 = greatest, [number of clusters] = least) were plotted in each column per experiment in a heatmap generated in *ggplot2* (Wickham, 2016). Clusters with similar phenotypic rankings among experiments were grouped visually into integrated phenotype groups (#1–6). Allometry between root anatomical phenotypes, shoot dry biomass, and nodal root number was modeled as a linear regression using the log_10_ transformed phenotype values. The slope of the regression (alpha), coefficient of determination (Adj. *R*^2^), and *p*-values were determined for selecting phenotype relationships. Coefficients were considered isometric at 0.33 ± 0.15 for linear phenotypes (nodal root number, percent aerenchyma) or 0.67 ± 0.15 for area phenotypes (RXSA, MXA).

## Results

### Drought stress response and root and shoot phenotypes differed among experiments

Sets of rice genotypes were grown under drought stress across multiple environments, and root phenotypes were measured to observe responses to stress and to identify phenotypic strategies that were positively associated with biomass accumulation across environments. In the greenhouse experiments RDP1 PSU and RILs PSU, drought was imposed on 2-week-old seedlings growing in PVC pipes filled with a sandy growth medium, and roots were sampled after 4 weeks of stress treatment. The RDP1 URRC rainout shelter experiment was direct-seeded onto sandy field soil in a cement trough, and drought was imposed at panicle initiation 5 weeks after emergence and lasted for 5 weeks before root sampling. In the RDP1 IRRI experiment, 3-week-old seedlings were transplanted into a puddled rainout shelter field, and drought was imposed 2 weeks after transplanting and extended for 2 weeks until sampling.

Root and shoot phenotypes varied among experiments (Supplementary Figure 1 and Supplementary Table 2). In the well-watered treatment, mean shoot biomass and tiller number were greatest in the RILs PSU experiment and least in the RDP1 IRRI experiment. Mean root cross-sectional area and living tissue area were greatest in the RDP1 URRC experiment, but percent aerenchyma area was least in the RDP1 URRC experiment compared to other experiments in well-watered conditions. Mean metaxylem vessel area and stele area were greatest in the RILs PSU experiment and least in the RDP1 IRRI experiment. In most experiments, shoot dry biomass, nodal root number, and root depth were more affected by treatment than by genotype, with the greatest effect sizes occurring in the RDP1 PSU experiment (based on *F*-value and η^2^, Table 2 and Supplementary Table 3). There was significant genotypic variation in shoot biomass in three of the four experiments, and significant (*p*-value ≤ 0.01) treatment × genotype interaction in one (RDP1 URRC). Genotypic variation in root phenotypes was greatest in the RDP1 URRC experiment (Table 2), which included the greatest number of genotypes (140) and the longest growth period before sampling (83 DAS).

**TABLE 2:** ANOVA table showing the effect of drought treatment, genotype, and interaction effects on nodal root anatomical traits, root architectural traits, and shoot traits within each experiment location.

RDP1 IRRI				RDP1 PSU		

*Root traits*	Treatment	Genotype	Treatment* Genotype	Treatment	Genotype	Treatment* Genotype
Root cross-sectional area	ns	ns	ns	67.9***	7.6***	ns
Living tissue area	4.1*	1.7^+^	ns	6.5*	4.0**	ns
Stele area	16.7***	3.7***	ns	10.3**	15.6***	2.3*
Cortical area	ns	ns	ns	76.4***	6.8***	ns
Aerenchyma area	ns	ns	ns	66.2***	4.4***	ns
Percent aerenchyma area	ns	ns	ns	13.8***	ns	ns
Mean metaxylem vessel area	33.4***	3.9***	ns	9.0**	17.6***	1.9^+^
Metaxylem vessel number	ns	2.4*	ns	6.2*	3.3**	ns
Crown root number	879.2***	2.8***	1.8***	176.4***	3.6**	2.6*
Root depth	28.5***	1.9^+^	2.4*	30.3***	ns	ns
*Shoot traits*						
Shoot dry biomass	216.2***	3.0***	1.2^+^	87.2***	3.7**	ns
Tiller number	27.8***	4.6***	ns	3.0^+^	ns	ns

**RDP1 URRC**				**RILs PSU**		

***Root traits***	**Treatment**	**Genotype**	Treatment* Genotype	**Treatment**	**Genotype**	Treatment* Genotype

Root cross-sectional area	85.0***	10.9***	6.5***	85.7***	1.8*	ns
Living tissue area	44.3***	9.9***	5.9***	75.0***	1.9**	1.4^+^
Stele area	1153.1***	34.4***	10.5***	20.4***	3.1***	ns
Cortical area	84.2***	10.3***	6.5***	93.3***	1.7*	ns
Aerenchyma area	90.9***	8.9***	6.3***	66.2***	1.4^+^	ns
Percent aerenchyma area	13.5***	5.8***	5.6***	ns	ns	ns
Mean metaxylem vessel area	1051.4***	38.1***	10.9***	44.1***	4.9***	ns
Metaxylem vessel number	9.7**	14.3***	4.9***	20.9***	2.4***	ns
Crown root number	629.4***	3.4***	2.1***	130.2***	ns	ns
Root depth	43.2***	3.0***	2.6***	14.9***	1.7*	ns
*Shoot traits*						
Shoot dry biomass	172.8***	2.9***	1.5**	52.0***	ns	ns
Tiller number	6.0*	5.3***	ns	37.1***	1.6*	ns

F-values and significance levels [p-value < 0.1+, 0.05*, 0.01**, 0.001***, not significant (ns)] are shown for each trait.

### Drought stress effects on root and shoot phenotypes across experiments

Across experiments, shoot dry biomass and tiller number generally decreased in response to drought stress (Supplementary Figure 1 and Supplementary Table 2). Plants in the RDP1 PSU experiment showed the greatest biomass (70%) and tiller number (30%) reductions in response to drought, while those in the RDP1 URRC experiment showed the least reductions (35% biomass, tiller number *ns*). In the RILs PSU experiment, drought reduced shoot dry biomass by 48% and tiller number by 35%, and in the RDP1 IRRI experiment, drought reduced shoot dry biomass by 50% and tiller number by 47%. Nodal root number reductions ranged from 84% in the RDP1 PSU experiment to 49% in the RDP1 URRC experiment.

**FIGURE 1:**
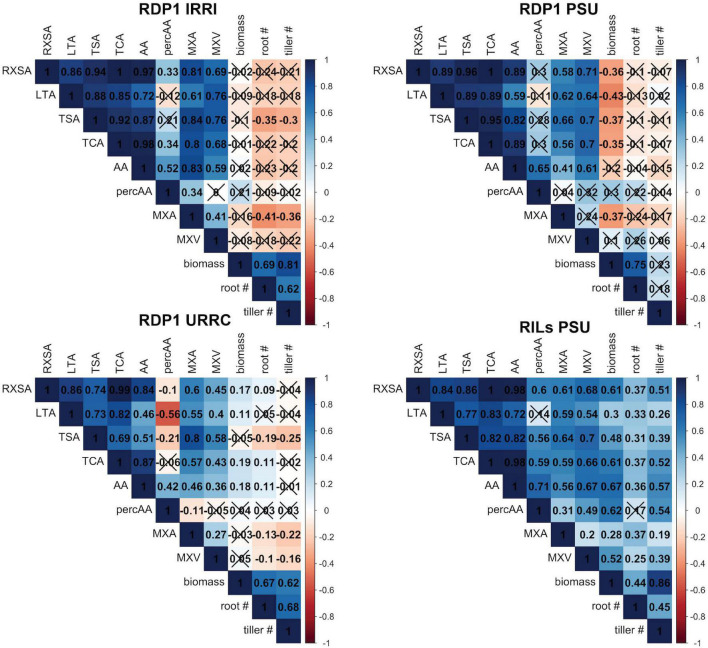
Correlation matrices by experiment. Correlation matrices of root anatomical traits, shoot dry biomass, nodal root number, and tiller number under drought stress within each experiment location. Each box shows the correlation coefficient, *r*, between each trait. X’s indicate that the correlation was not significant at the α = 0.1 level. See Table 1 for phenotype abbreviations.

The effects of drought on root anatomy varied among experiments, as did the treatment × genotype interaction (Table 2, Supplementary Figure 1, and Supplementary Table 2). In the RDP1 PSU and the RILs PSU experiments, median root cross-sectional area was reduced by drought, and since root diameter was correlated with other anatomical phenotypes except percent aerenchyma (Figure 1), these anatomical phenotypes were also affected. In the RDP1 IRRI and the URRC experiments, drought resulted in greater stele and mean metaxylem vessel areas, while in the RDP1 PSU and RILs PSU experiments, there was no effect or a reduction in stele and median metaxylem vessel areas with drought. Plants in the RDP1 URRC experiment had the strongest treatment × genotype interactions, which were highly significant for every phenotype measured.

### Relationships between root phenotypes and shoot biomass were inconsistent

Root anatomical phenes were positively correlated with each other, but correlations with shoot biomass and tiller number varied among experiments, ranging from positive to insignificant to negative (Figure 1). These relationships were somewhat stronger under drought than in well-watered conditions (Figure 1 and Supplementary Figure 2). In the RILs PSU experiment, all root anatomical phenes were positively correlated with shoot dry biomass and tiller number, while in the RDP1 PSU experiment, most anatomical root phenes were negatively correlated with shoot dry biomass and none correlated with nodal root or tiller number. We were, therefore, unable to use simple correlations to discover how root anatomical phenes could contribute to better performance with drought stress.

### Some root phenotypes were associated with best and worst performing groups under drought

**FIGURE 2:**
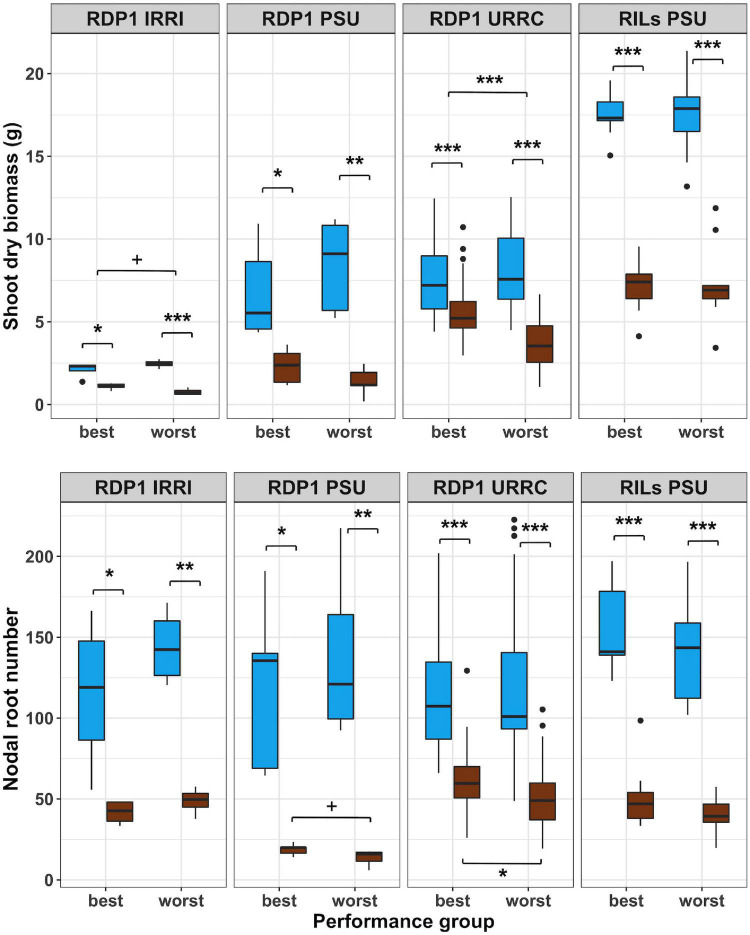
Boxplots of shoot phenotypes of best and worst groups. Shoot dry biomass and nodal root number under well-watered (blue) and drought (brown) treatments in best and worst performing groups within each experiment location. Genotypes with mean biomass greater than one standard deviation from the mean under well-watered conditions were filtered out, and the remaining genotypes were grouped into best and worst groups based on 50% top and bottom biomass plasticity, respectively. Significance levels (*p*-value < 0.1+, 0.05*, 0.01^**^, 0.001^***^) are shown for *t*-tests between treatments and performance groups within each experiment.

In another approach to determine the potential value of root anatomical phenes for drought tolerance, we identified the best and worst performing groups within each experiment using bulked segregant analysis of phenotypes based on the reduction in shoot biomass by drought for each experiment. As a result of this filtering method, the best groups had similar biomass compared to the worst groups in the well-watered treatment, but the best groups had smaller biomass reductions under drought than the worst groups (Figure 2 and Supplementary Table 4). The best and worst groups also showed contrasts in root architectural phenotypes depending on the experiment. Nodal root number was reduced by drought in all groups, but in the RDP1 PSU and URRC experiments, the best groups had significantly more nodal roots than the worst groups in the drought treatment. In the greenhouse experiments, where maximum root depth was measured, RDP1 PSU and RILs PSU, maximum root depth was greater in the best groups compared to the worst groups under drought treatment (Supplementary Table 4).

**FIGURE 3:**
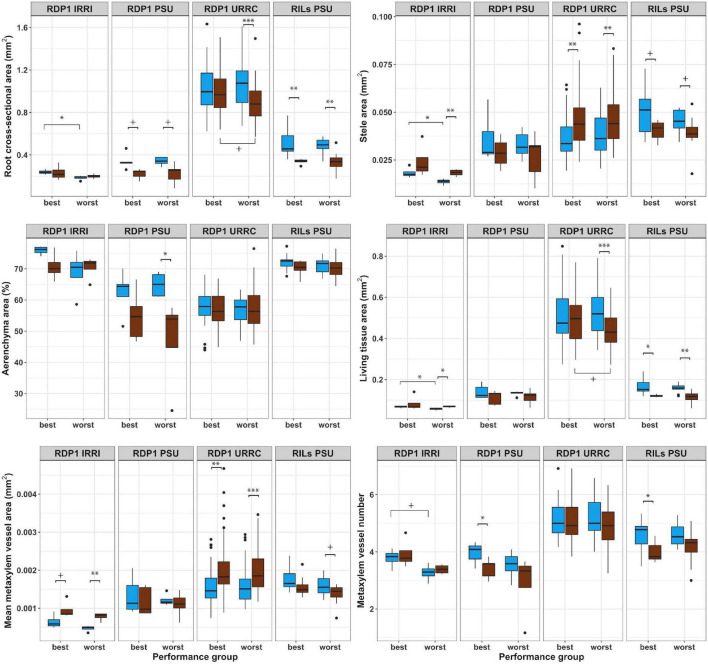
Boxplots of root anatomical phenotypes of best and worst groups. Root anatomical phenotypes under well-watered (blue) and drought (brown) treatments in best and worst performing groups within each experiment location. Significance levels (*p*-value < 0.1+, 0.05*, 0.01^**^, 0.001^***^) are shown for *t*-tests between treatments and performance groups within each experiment.

Root anatomical phenotypes and their responses to drought treatment differed between best and worst groups in a few cases (Figure 3 and Supplementary Table 4). In the rainout shelter experiments, RDP1 IRRI and URRC, living tissue areas exhibited plasticity in response to drought treatment in the worst group, while the best group was not plastic. In the RDP1 URRC experiment, root cross-sectional area, cortical area, and living tissue area were greater in the best group compared to the worst group under drought treatment. In the greenhouse experiments, RDP1 PSU and RILs PSU, there were no differences in root anatomical phenotypes between the best and worst groups in either treatment.

### Integrated root phenotypes under drought were common among experiments

Since root phenotypes act in concert to regulate the performance of crop, we examined how integrated root phenotypes are related to performance under drought. First, individuals in each experiment were grouped into clusters based on the similarity of root phenotypes under drought (Supplementary Table 5), which resulted in four clusters in the RDP1 IRRI experiment, three clusters in the RDP1 PSU experiment, five clusters in the RDP1 URRC experiment, and four clusters in the RILs PSU experiment. Silhouette width plots show the similarity among individuals within clusters, and plots of the first two principal components show the separation among clusters within each experiment (Supplementary Figure 3).

**FIGURE 4:**
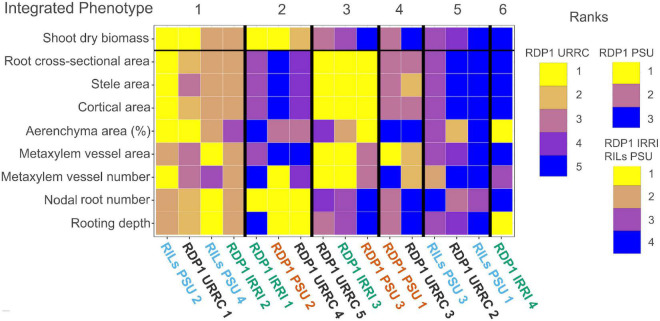
Heatmap of drought cluster phenotype rankings and integrated phenotypes. Summary heatmap of drought phenotype rankings among experiments. Median trait values of each cluster were ranked greatest (yellow) to least (blue) compared to the other cluster trait medians within each experiment. Each column represents phenotype ranks from one cluster. Clusters with similar trait rankings across experiments were grouped by integrated phenotype.

Next, phenotypes of clusters within experiments were ranked by phene values for each of the shoot and root phenotypes measured. For example, RDP1 PSU plants were grouped into three clusters by PAM clustering of root phenotypes. RDP1 PSU cluster 2 individuals had a mean shoot dry biomass under drought of 3.52 g (Supplementary Table 5) and were thus ranked first (1, yellow, Figure 4) in biomass among RDP1 PSU clusters; RDP1 PSU cluster 1 individuals had a mean shoot dry biomass under drought of 2.427 g and were thus ranked second (2, mauve) in biomass among RDP1 PSU clusters; RDP1 PSU cluster 3 individuals had a mean shoot dry biomass under drought of 1.472 g and were thus ranked third (3, dark blue) in biomass among RDP1 PSU clusters. Shoot biomass varied among clusters in three of the four experiments in the drought treatment (Supplementary Figure 4A), while shoot biomass did not vary among clusters in any of the experiments in the well-watered treatment (Supplementary Figure 4B).

By visually grouping similar phenotype rankings among clusters, we identified six integrated phenotypes under drought treatment across all experiments, each composed of clusters from several experiments (Figure 4). Integrated phenotypes #1–2 had the greatest relative shoot biomass values under drought, while integrated phenotypes #3–6 did not perform as well. Clusters within integrated phenotype #1 showed high rankings for most root anatomical phenotypes, including both root cross-sectional and percent aerenchyma area, though some clusters had lower rankings for metaxylem vessel size, metaxylem vessel number, stele area, and percent aerenchyma. All integrated phenotype #1 clusters had many, deep nodal roots, and most clusters showed relatively large shoot dry biomass under drought treatment in their respective experiments. Clusters within integrated phenotype #2 had low root anatomical phenotype rankings, though some had moderate values for percent aerenchyma area. Most integrated phenotype #2 clusters had many, deep nodal roots and performed well under drought. Clusters comprising integrated phenotype #3 had mostly large root anatomical phenotype rankings with few nodal roots at shallow to moderate depths. Two clusters within this integrated phenotype had moderate to small shoot biomasses in their respective experiments (RDP1 PSU and URRC). Clusters within integrated phenotype #4 had moderate root cross-sectional area, stele area and cortical area, high metaxylem vessel area, and low percent aerenchyma area rankings with moderate to few nodal roots at shallow to moderate depth. The two clusters within integrated phenotype #4 showed relatively poor performance in terms of shoot dry biomass in their respective experiments (RDP1 PSU and URRC). Clusters comprising integrated phenotype #5 had small root anatomical phenotype rankings, few shallow nodal roots, and small shoot biomass under drought. The final cluster from the RDP1 IRRI experiment (integrated phenotype #6) did not have phenotype similarities to any other clusters, as it had a large percent aerenchyma area, few metaxylem vessels, few but deep nodal roots, and low shoot biomass ranking.

Since the plant size (shoot biomass) ranged considerably among experiments, which could affect phenotypes, such as nodal root number and root diameter, we calculated the allometric partitioning coefficients of the integrated phenotypes (Table 3). Integrated phenotype #6 had too few individuals so is not included here. Most integrated phenotypes had hyperallometric relationships between nodal root number and shoot biomass, i.e., nodal root number scaled with shoot biomass to a proportionately greater extent than expected. With drought, the poor-performing integrated phenotype #5 lacked significant allometric relationships between shoot biomass and nodal root number, while the top four integrated phenotypes maintained similar or greater (#4) coefficients compared with well-watered plants, i.e., drought did not have much effect on the balance between shoot growth and nodal root number. Root cross-sectional area displayed isometric scaling with shoot biomass under well-watered conditions, but the top-ranked integrated phenotypes increased their coefficients under drought to the extent that two of them became hyperallometric. Metaxylem vessel area vs. shoot biomass displayed hyperallometric coefficients in the top three integrated phenotypes under both treatments, with increased positive scaling under drought. Metaxylem vessel area was hyperallometric with root cross-sectional area in all the integrated phenotypes under drought, and in most of them in well-watered conditions. Percent aerenchyma tended to have either insignificant or hypoallometric relationships with shoot biomass, and strong hypoallometry was most pronounced in integrated phenotype #2 under drought.

**TABLE 3:** Allometric partitioning coefficients (alpha) describing the slope of the natural logarithm of root traits vs. the natural logarithm of shoot dry biomass or root cross-sectional area.

	Integrated phenotype group	Well-watered				Drought			
Trait		Adj. *R*^2^	Alpha	*p*-value	*n*	Adj. *R*^2^	Alpha	*p*-value	*n*
Nodal root number	1	**0.09**	**0.90**	**0.011**	**59**	**0.12**	**0.98**	**0.004**	**59**
vs. shoot biomass	2	**0.16**	**0.70**	**0.006**	**39**	**0.13**	**0.65**	**0.015**	**39**
	3	**0.5**	**1.58**	**<0.001**	**27**	**0.48**	**1.11**	**<0.001**	**27**
	4	**0.21**	**0.42**	**0.021**	**21**	**0.48**	**0.83**	**<0.001**	**21**
	5	**0.09**	**0.57**	**0.021**	**50**	0.02	-0.18	0.192	50
	6	0.68	0.73	0.262	3	0.44	0.89	0.353	3
RXSA	1	**0.29**	**0.71**	**<0.001**	**59**	**0.27**	**0.90**	**<0.001**	**59**
vs. shoot biomass	2	**0.39**	**0.60**	**<0.001**	**39**	**0.45**	**0.71**	**<0.001**	**39**
	3	**0.42**	**0.75**	**<0.001**	**27**	**0.58**	**1.09**	**<0.001**	**27**
	4	0.06	-0.17	0.143	21	0.04	0.17	0.204	21
	5	**0.27**	-**0.59**	**<0.001**	**50**	**0.09**	-**0.30**	**0.019**	**50**
	6	<0.01	-1.47	0.508	3	0.26	4.82	0.417	3
MXA	1	**0.61**	**1.28**	**<0.001**	**59**	**0.46**	**2.14**	**<0.001**	**59**
vs. shoot biomass	2	**0.28**	**0.82**	**<0.001**	**39**	**0.12**	**0.94**	**0.016**	**39**
	3	**0.42**	**1.23**	**<0.001**	**27**	**0.56**	**1.51**	**<0.001**	**27**
	4	0.07	-0.38	0.124	21	<0.01	0.21	0.476	21
	5	**0.08**	**0.58**	**0.027**	**50**	0.03	-0.48	0.106	50
	6	0.22	-2.06	0.431	3	0.02	-2.67	0.493	3
MXA	1	**0.47**	**0.89**	**<0.001**	**59**	**0.56**	**1.39**	**<0.001**	**59**
vs. RXSA	2	**0.71**	**1.35**	**<0.001**	**39**	**0.65**	**1.92**	**<0.001**	**39**
	3	**0.76**	**1.44**	**<0.001**	**27**	**0.83**	**1.29**	**<0.001**	**27**
	4	**0.61**	**1.66**	**<0.001**	**21**	**0.79**	**1.95**	**<0.001**	**21**
	5	<0.01	0.27	0.263	50	**0.25**	**1.19**	**<0.001**	**50**
	6	0.97	1.25	0.077	3	<0.01	-0.09	0.91	3
% aerenchyma	1	<0.01	-0.25	0.772	59	<0.01	-0.60	0.542	59
vs. shoot biomass	2	**0.43**	-**3.41**	**<0.001**	**39**	**0.4**	-**4.03**	**<0.001**	**39**
	3	**0.22**	-**2.63**	**0.007**	**27**	**0.17**	-**2.36**	**0.018**	**27**
	4	<0.01	-0.19	0.735	21	<0.01	0.60	0.339	21
	5	**0.71**	**3.19**	**<0.001**	**50**	**0.18**	**1.52**	**0.001**	**50**
	6	<0.01	0.72	0.964	3	<0.01	1.92	0.732	3

Coefficients are considered isometric at 0.33 + 0.15 for linear traits (nodal root number, percent aerenchyma) or 0.67 + 0.15 for area traits (RXSA, MXA). Bold values indicate significant allometric relationships. The coefficient of determination (Adj. R2) and p-values are shown for each relationship.

## Discussion

All phenes exist as part of one organism, and multiple root phenes interact to produce synergistic effects on soil resource capture (Ma et al., 2001; York et al., 2013; Miguel et al., 2015; Klein et al., 2020; Rangarajan and Lynch, 2021). In this study, integrated root phenotypes, i.e., specific combinations of root phenes, were better associated with performance (greater biomass) under drought within and across experiments compared to either individual phenes or those grouped by phenotypic bulked segregant analysis. Correlations of individual root traits and phenes with shoot biomass were weak and inconsistent across experiments, and bulked segregant analysis did not successfully explain differences in biomass accumulation with drought. PAM clustering enabled phenotypic grouping based on root architectural and anatomical phenes under drought, allowing us to elucidate the effects of integrated phenotypes on the performance of drought across experiments, even given the differences in plant age and developmental stage among experiments. Integrated root phenotypes are often better associated with performance under drought and/or low fertility than individual root phene states (York and Lynch, 2015; Sandhu et al., 2016; Strock et al., 2019; Klein et al., 2020), and *in silico* studies have shown that multiple integrated phenotypes improve the performance of plant under abiotic stresses, such as low P and N (Ma et al., 2001; Dunbabin et al., 2003; Postma and Lynch, 2011; Dathe et al., 2016; Rangarajan et al., 2018).

One phenotype that was consistently associated with good performance under drought stress was deep rooting. Integrated phenotypes #1 and #2 exhibited greater rooting depth and less biomass loss with drought stress. The only exception to this was the cluster RDP1 IRRI 1, which had the smallest plants in both irrigated and drought conditions (Figure 4 and Supplementary Figure 4). Rooting depth in the IRRI experiment was assessed by root length distribution in soil cores. All clusters in that experiment distributed 10–21% of root length below 20 cm in the drought treatment (Supplementary Table 5), and only one soil core in the entire experiment failed to include any root length below 20 cm, so it was likely that nearly all plants were able to access some water at depth. In the other experiments, all the clusters with deep roots, measured directly in the PSU experiments and by root angle in the URRC experiment, had top-ranked performance under drought.

Studies have shown that deeper rooting increases drought tolerance in rice by providing plants with access to soil moisture in deep soil strata (Uga et al., 2008, 2013; Henry et al., 2011; Arai-Sanoh et al., 2014). Deeper rooting is facilitated by steeper root angles (Uga et al., 2013), but additional phenes can also contribute to deep rooting. We hypothesized that phenes conferring reduced root metabolic cost per unit root length, such as smaller root diameter and/or more aerenchyma, would improve drought tolerance in rice as they have in other crops (Lynch, 2013, 2015) by reducing the cost of soil exploration. Integrated phenotype group #2 was consistent with this idea, exhibiting small root cross-sectional area, deep rooting, and greater shoot biomass under drought. Phenotypes related to reduced metabolic cost per unit root length should be more important when root number is large. All three component clusters in this integrated phenotype were top-ranked for the number of nodal roots relative to others in their experiments. Integrated phenotype #2 appears to have been successful in minimizing biomass loss during drought by prioritizing investment in nodal root number and length and limiting investment in root diameter.

The other integrated phenotype with the best performance (shoot biomass rank) showed very different anatomical phenotypes, i.e., integrated phenotype #1 had large diameter roots in contrast to the smaller diameter roots characteristic of integrated phenotype #2 (Figure 4 and Supplementary Table 5). Clusters within integrated phenotype #1 ranked high for the number and depth of nodal roots, root cross-sectional area, and percent aerenchyma area. Root cross-sectional area became hyperallometric under drought in integrated phenotype #1, suggesting that there were advantages to larger root diameters in the context of this integrated phenotype. Greater aerenchyma area presumably reduced the metabolic cost of these large diameter nodal roots (Fonta et al., 2022), permitting both larger numbers of nodal roots and greater root depth in this integrated phenotype.

Integrated phenotype #3 had notably greater biomass reduction under drought than integrated phenotypes #1 and #2. The clusters within integrated phenotype #3 had even larger diameter roots than those comprising integrated phenotype #1, and, like the better performing integrated phenotypes, nodal root number was hyperallometric relative to shoot biomass under drought (Table 3). However, nodal roots in integrated phenotype #3 were fewer and shallower (Figure 4). Very large diameter roots are metabolically costly, even with a large amount of aerenchyma, such as found in cluster RDP1 PSU 3, and this cost could have diminished lateral root formation and elongation of all root classes, preventing adequate water and nutrient uptake under stress. The fact that all clusters comprising integrated phenotype #3 had the largest diameter roots relative to other integrated phenotypes within their experiments suggests that there is a limit to the ability of rice plants to compensate for the greater cost of large diameter roots.

Clusters within integrated phenotype #4 had moderate diameter roots but very little aerenchyma and produced relatively few nodal roots that ranked low for root depth. Poor aerenchyma formation may have been responsible for the weaker performance of integrated phenotype #4. Clusters within integrated phenotype #5 and #6 had relatively small diameter roots but did not perform well, perhaps because they did not produce adequate nodal root number and/or depth to sustain growth under drought. Many other phenes related to drought responses that we did not measure, such as stomatal density, leaf area, leaf water potential, lateral root growth, and root hair formation, may have played additional roles in these clusters in conjunction with phenes related to root metabolic cost. In all, the best-performing integrated phenotypes maintained large root number and greatest root depth through reduced root metabolic cost, achieved *via* abundant aerenchyma or smaller diameter roots.

One hypothesis that was not supported by these experiments was the idea that fewer nodal roots would increase root depth, and therefore, improve performance under drought. In maize, fewer nodal roots improved yield under drought (Lynch, 2015; Gao and Lynch, 2016) and suboptimal nitrogen availability (Saengwilai et al., 2014b) by conserving metabolic energy to promote deeper rooting by fewer roots. In these experiments, the top-performing integrated phenotypes #1 and #2 had many nodal roots, with every cluster comprising those integrated phenotypes ranking above the median value for nodal root number. Under drought, all four of the top-performing integrated phenotypes maintained hyperallometry of nodal root number scaled with biomass (Table 3), indicating that they continued to invest resources in nodal root number disproportionately to biomass, but the integrated phenotypes with weaker performance under drought were unable to produce as many nodal roots as the best-performing integrated phenotypes. Compared with maize, rice has many nodal roots, with over 100 nodal roots under well-watered conditions in all of our experiments (Supplementary Figure 1), while maize has 10–30 nodal roots in greenhouse experiments and up to 80 nodal roots in the field at flowering (Gao and Lynch, 2016). The diameter of maize nodal roots is also much larger, with 2–7 times the cross-sectional area of rice nodal roots at comparable stages of development (Burton et al., 2015; Yang et al., 2019; Klein et al., 2020), and therefore, more metabolically expensive per unit length. Rice could, therefore, be considered to already have a “many, cheap” nodal root strategy relative to maize and benefits less from reducing nodal root numbers.

### Best integrated phenotypes have contrasting metaxylem phenotypes

We expected that smaller metaxylem vessels would benefit plants under drought stress by reducing axial hydraulic conductance to conserve soil moisture and prevent vessel cavitation and collapse. We observed this phenotype in integrated phenotype #2, but integrated phenotype #1, which had similar overall maintenance of shoot biomass with drought, had moderate to large vessels. These two integrated phenotypes showed contrasts in metaxylem vessel phenotypes similar to those in root diameter (Figure 4 and Supplementary Table 5), which was unsurprising since metaxylem size and number tended to scale positively with root cross-sectional area in all the experiments (Figure 1).

Comparing integrated phenotypes #1 and #3 is useful since these groups had the largest diameter nodal roots but very different performances in biomass rankings under drought. These integrated phenotypes had the strongest hyperallometric scaling of median metaxylem vessel area with shoot biomass and the greatest increase in scaling coefficient with drought, suggesting that the phenotypes with larger diameter roots were characterized by high axial hydraulic conductance to support larger shoots. The difference in performance between these integrated phenotypes could have resulted from differences in nodal root number and rooting depth, since clusters with integrated phenotype #1 were able to grow many deep roots but clusters with integrated phenotype #3 did not. These integrated phenotypes could be characterized as having a “water-spending strategy,” relying on the continued availability of deep water to avoid drought-induced biomass reduction. In cases, such as integrated phenotype #3, where large diameter roots with high axial conductance fail to reach deep water, the result is a weaker performance with drought stress.

Integrated phenotype #2 had metaxylem phenotypes most consistent with our hypothesis, i.e., very low ranking for metaxylem vessel area. Two of the three clusters with this integrated phenotype also had low-ranking metaxylem vessel number, which we expected to be larger to partially compensate for the smaller vessel diameter. In those two clusters, the large number of nodal roots may have compensated for the reduced axial conductance per root. The cluster RDP2 PSU 2 within integrated phenotype #2 was closest to our proposed optimal phenotype. It had the “more numerous, narrower” metaxylem vessel phenotype proposed to provide adequate axial conductance in nodal roots with minimal risk of cavitation (York and Lynch, 2015; Sandhu et al., 2016; Strock et al., 2019; Klein et al., 2020), combined with small diameter nodal roots, which are expected to have low metabolic cost. These phenes would have contributed to the development of many, deep nodal roots resulting in top-ranked biomass accumulation within that experiment.

### Multiple integrated phenotypes perform well within the same environments

We hypothesized that multiple, distinct integrated phenotypes would be beneficial in the same environment, as previous studies have shown in field-grown plants (York and Lynch, 2015; York et al., 2015; Sandhu et al., 2016; Strock et al., 2019; Klein et al., 2020) and *in silico* (Ma et al., 2001; Dunbabin et al., 2003; Dathe et al., 2016; Rangarajan et al., 2018). In these experiments, we found representative clusters from each experiment in at least one of the best-performing but contrasting integrated phenotypes #1 and #2 (Figure 4 and Supplementary Table 5). There are, therefore, multiple solutions to the problem of acquiring adequate moisture under drought. Where greater root diameter is an advantage, e.g., in hard soils, steep-angled, large diameter nodal roots with abundant aerenchyma and high axial hydraulic conductance could acquire deeper soil moisture, while in softer soils, many small diameter roots with low axial hydraulic conductance could achieve the same end. Phenotypic plasticity was not considered separately from individual phene states during the creation of the integrated phenotypes in this study. Alteration of root phenes in response to drought could be important, particularly when drought occurs during vegetative development, and there is time for root phenotypic changes to influence water acquisition. These options increase the potential for plant breeders to optimize drought tolerance for a variety of production environments.

## Conclusion

Drought stress is a major constraint to rice production, and root phenotypes that promote growth under drought could help offset yield losses. Root anatomical, root architectural, and shoot phenes are interdependent and vary by environment, and their responses to drought all play a role in the performance of plant. Multiple integrated root phenotypes can be successful in a given environment if they ultimately promote efficient, sufficient uptake and transport of water and nutrients. Our results using diverse accessions (including genotypes from a diversity panel and RILs) and growth environments showed that no phenotype in isolation contributed significantly to drought tolerance in all situations, and different integrated root phenotypes were effective within and across environments. The best-integrated phenotypes in these experiments had many deep roots, but there were multiple combinations of other phenotypes that permitted good plant growth under drought. This study did not include measurements of shoot phenotypes, which are also important for performance under drought stress. Shoot phenotypes may interact with root phenotypes in interesting and important ways. The value of particular integrated phenotypes will vary depending on environmental constraints. Given the genetic variation for all root phenotypes and their plasticity, additional integrated phenotypes of value in drought environments may be present in existing germplasm.

## Data availability statement

The original contributions presented in the study are included in the article/Supplementary material, further inquiries can be directed to the corresponding author/s.

## Author contributions

JF and KB conceived and designed the research with the assistance of JL. JF performed greenhouse experiments at Penn State. PV performed field experiments at URRC. AH performed field experiments at IRRI. JF contributed to sampling and phenotyping. JF and KB analyzed data with input from JL and AH. JF and KB wrote the manuscript with contributions and approval from JL, AH, and PV. All authors contributed to the article and approved the submitted version.
