# Supplementary material for: Many paths to one goal: Identifying integrated rice root phenotypes for diverse drought environments
Source: Front Plant Sci. 2022 Aug 22;13:959629. doi: 10.3389/fpls.2022.959629 (PMC9441928; doi:10.3389/fpls.2022.959629)
Supplement: Supplementary file 2 [file Image_1.pdf]

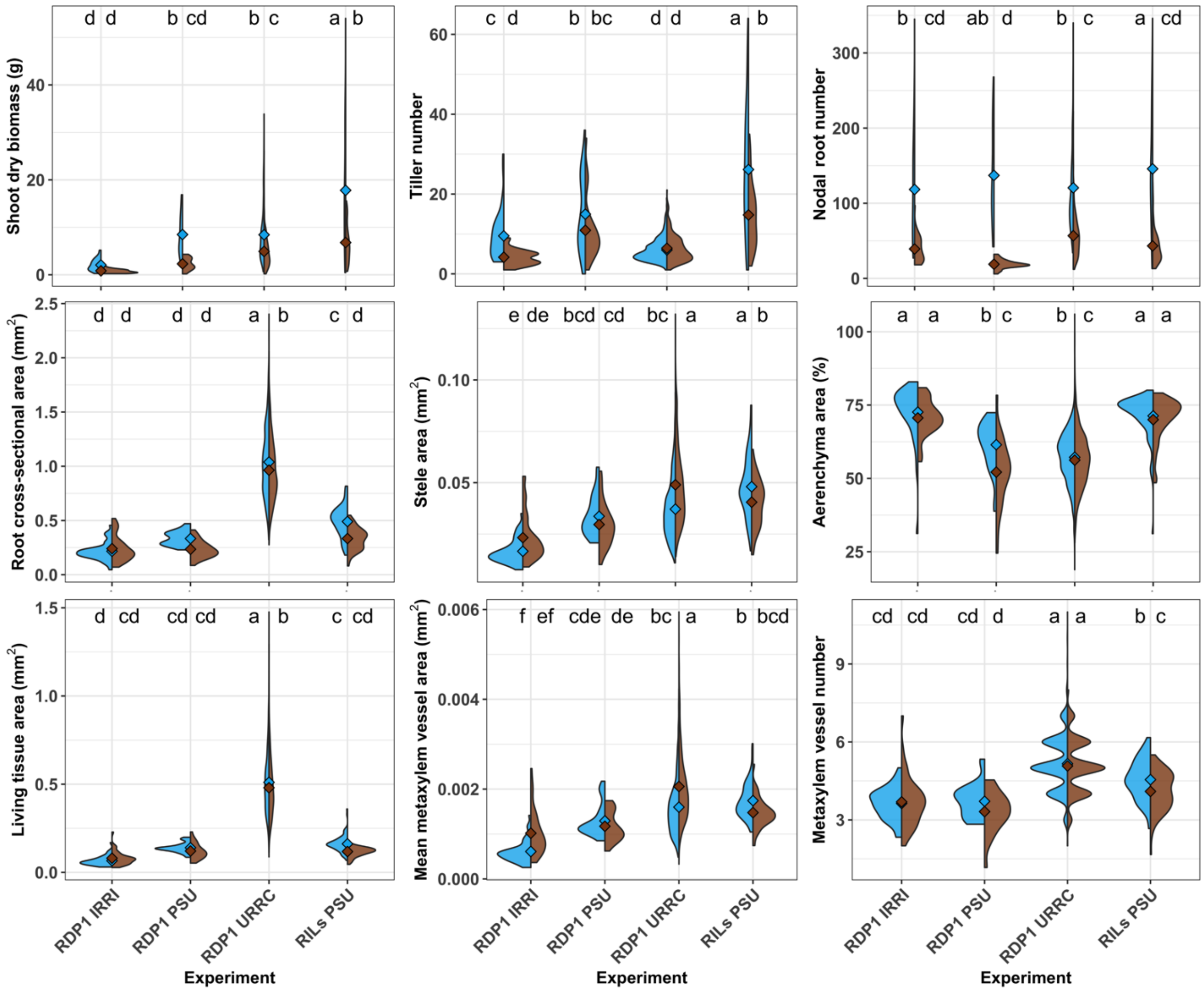

Supplemental Figure 1: Phenotype distributions within treatments and experiments. Shoot and root phenotypes under well-watered (blue) and drought (brown) treatments within each experiment. Diamonds represent the median value of the trait. Letters indicate significance groups ($\alpha = 0.1$) determined by multiple comparisons tests using Tukey's method.
