# Supplementary material for: Many paths to one goal: Identifying integrated rice root phenotypes for diverse drought environments
Source: Front Plant Sci. 2022 Aug 22;13:959629. doi: 10.3389/fpls.2022.959629 (PMC9441928; doi:10.3389/fpls.2022.959629)
Supplement: Supplementary file 3 [file Image_2.pdf]

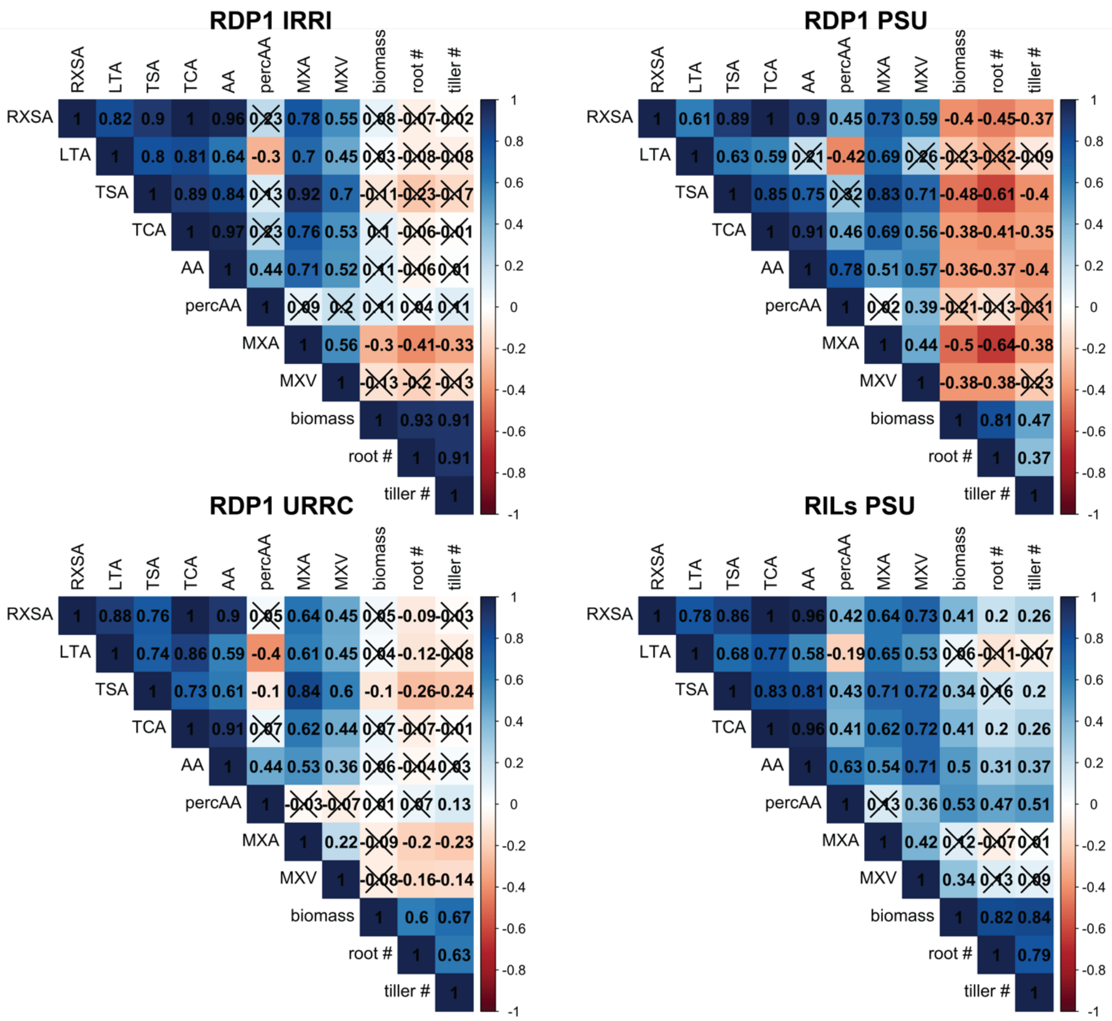

Supplemental Figure 2: Correlation matrix in combined locations. Correlation matrices of root anatomical traits, shoot dry biomass, nodal root number and tiller number under well-watered treatment within experiments. Each box shows the correlation coefficient, *r*, between each trait. X's indicate that the correlation was not significant below $\alpha = 0.1$. See Table 1 for phenotype abbreviations.
