# Supplementary material for: Many paths to one goal: Identifying integrated rice root phenotypes for diverse drought environments
Source: Front Plant Sci. 2022 Aug 22;13:959629. doi: 10.3389/fpls.2022.959629 (PMC9441928; doi:10.3389/fpls.2022.959629)
Supplement: Supplementary file 4 [file Image_3.pdf]

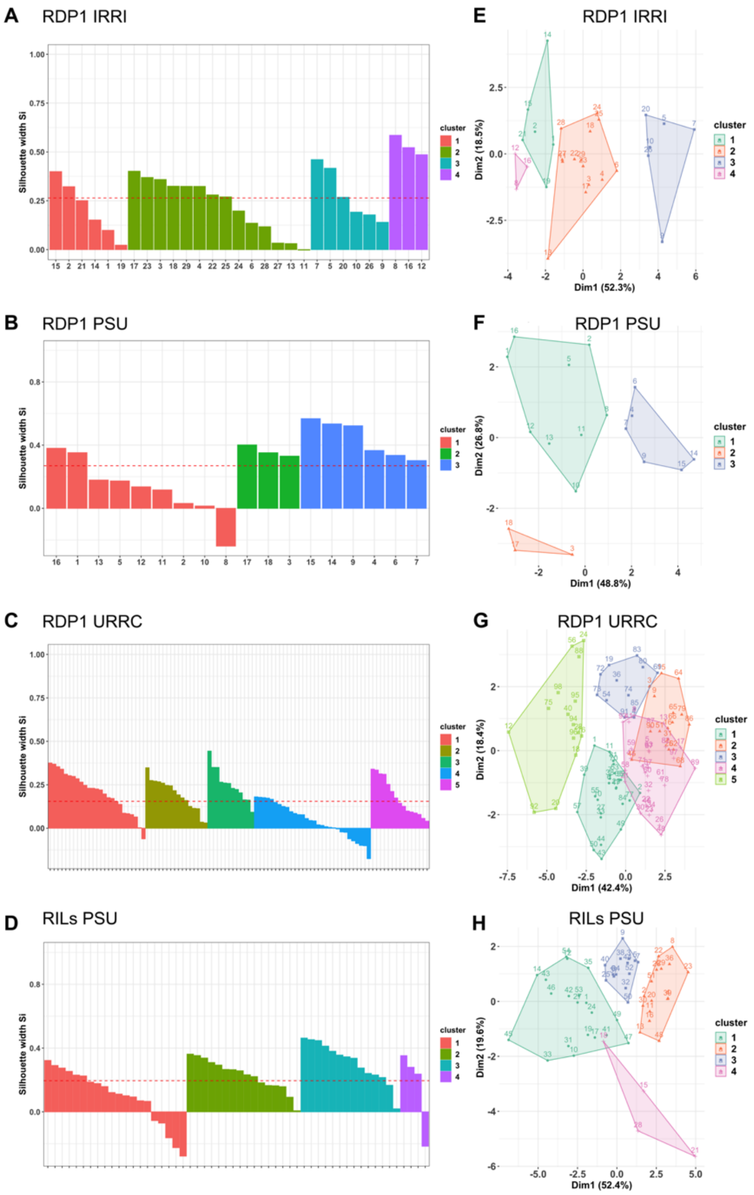

Supplemental Figure 3: Silhouette and PCA plots for drought clusters. Partitioning-around-medoids (PAM) clustering of individuals under drought stress based on root anatomical and architectural phenotypes under drought. Silhouette plots (A-D) show the similarity of individuals within clusters. Silhouette widths show the similarity of an individual to all other individuals within the same cluster, where 1 indicates perfect cohesion of the individual to others in the clusters and -1 indicates total dissimilarity. Average silhouette widths across all individuals (red dotted line) are 0.26 (A), 0.27 (B), 0.15 (C) and 0.20 (D). Principle component plots (E-H) show the loadings of the first two principle components (Dim1 and Dim2) separating the clusters of individuals. The percentage of variation explained by each principle component is noted in the axis label in parenthesis.
