# Supplementary material for: Many paths to one goal: Identifying integrated rice root phenotypes for diverse drought environments
Source: Front Plant Sci. 2022 Aug 22;13:959629. doi: 10.3389/fpls.2022.959629 (PMC9441928; doi:10.3389/fpls.2022.959629)
Supplement: Supplementary file 5 [file Image_4.pdf]

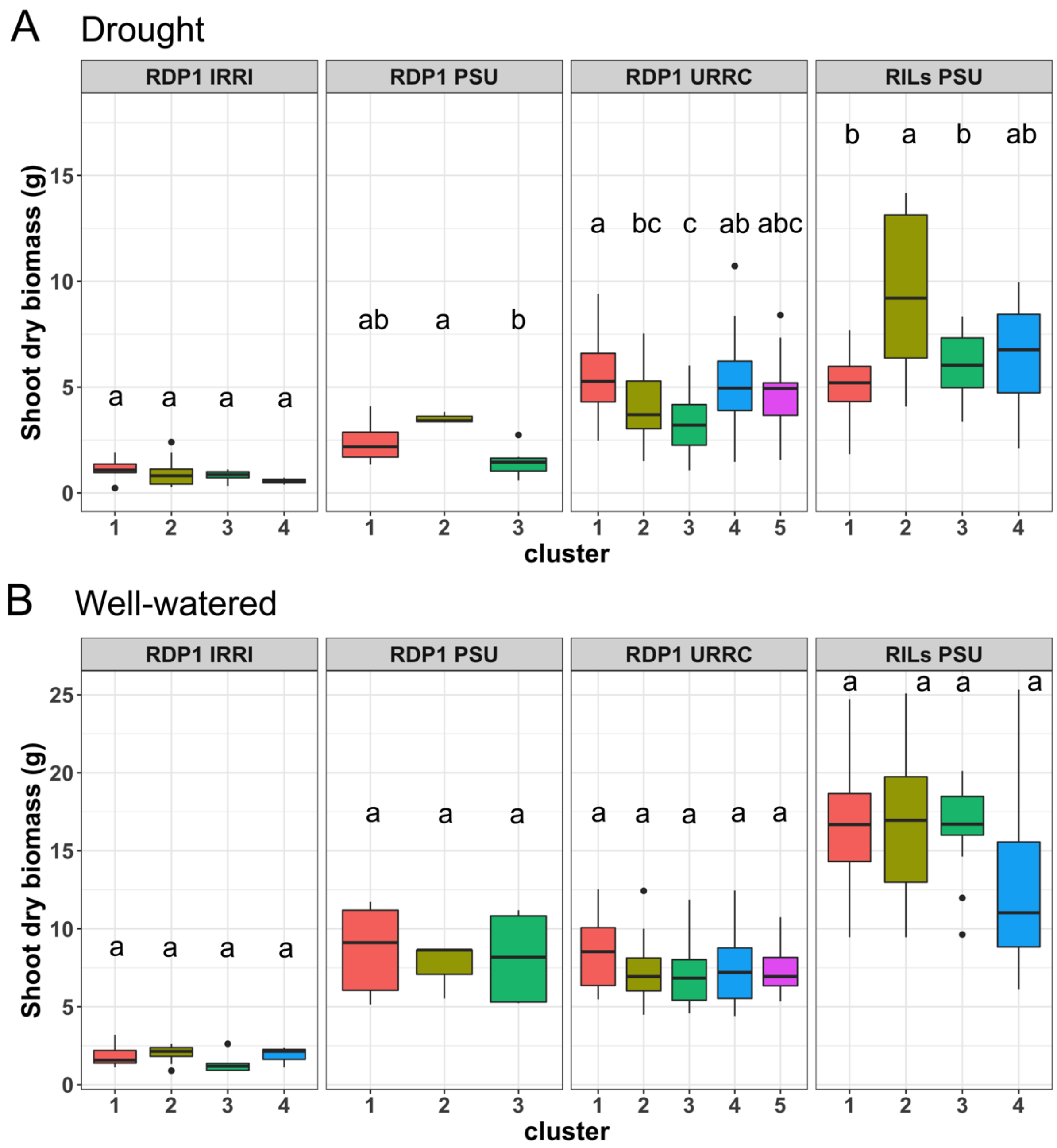

Supplemental Figure 4: Biomass in drought and well-watered treatment across clusters. Shoot dry biomass in A) drought and B) well-watered conditions of individuals within each cluster in each experiment. Letters indicate significance groups ($\alpha = 0.1$) determined by multiple comparisons tests using Tukey's method.
